# Supplementary material for: Empirical methods for controlling false positives and estimating confidence in ChIP-Seq peaks
Source: BMC Bioinformatics. 2008 Dec 5;9:523. doi: 10.1186/1471-2105-9-523 (PMC2628906; doi:10.1186/1471-2105-9-523)
Supplement: Additional File 1 — A variety of html documents from the USeq web site detailing the available applications, their best usage, output file type descriptions, command line menus, etc. [file 1471-2105-9-523-S1.zip › USeqUserGuides/JavaDoc.zip!/JavaDoc/util/bio/wrappers/package-summary.html]

util.bio.wrappers


|  |  |  |
| --- | --- | --- |
| **Overview** \| **Package** \| Class \| **Use** \| **Tree** \| **Deprecated** \| **Index** \| **Help** | |  |
| **PREV PACKAGE**   **NEXT PACKAGE** | **FRAMES**    **NO FRAMES**     **All Classes** |  |


---

## Package util.bio.wrappers

Wrappers for other bio applications.

**See:**
  
          **Description**

| **Class Summary** | |
| --- | --- |
| **BL2Seq** | This is a bit of a hack from the SimpleBlast, expand as needed. |
| **BL2SeqHit** | BL2Seq result container. |
| **Blast** | Template to fire and fetch results from the ncbi bl2seq program. |
| **BlastResult** | Information related to a blast results. |
| **Primer3Pick** | Container for sequence used in picking primers with Primer3. |
| **Primer3Wrapper** | This app wraps the command line primer3 app to make it useful for picking qPCR primers. |
| **Segment** | This wraps Huber's segment() function in his 'tilingArray' R package. |
| **SimpleBlast** | Wrapper for bl2seq blast. |

## Package util.bio.wrappers Description

Wrappers for other bio applications.

---


|  |  |  |
| --- | --- | --- |
| **Overview** \| **Package** \| Class \| **Use** \| **Tree** \| **Deprecated** \| **Index** \| **Help** | |  |
| **PREV PACKAGE**   **NEXT PACKAGE** | **FRAMES**    **NO FRAMES**     **All Classes** |  |


---
